# Recovery Analysis of Sequentially Irradiated and NBT-Stressed VDMOS Transistors

**DOI:** 10.3390/mi16010027

**Published:** 2024-12-28

**Authors:** Snežana Djorić-Veljković, Emilija Živanović, Vojkan Davidović, Sandra Veljković, Nikola Mitrović, Goran Ristić, Albena Paskaleva, Dencho Spassov, Danijel Danković

**Affiliations:** 1 Faculty of Civil Engineering and Architecture, University of Niš, Aleksandra Medvedeva 14, 18104 Niš, Serbia; 2 Faculty of Electronic Engineering, University of Niš, Aleksandra Medvedeva 4, 18000 Niš, Serbia; vojkan.davidovic@elfak.ni.ac.rs (V.D.); sandra.veljkovic@elfak.ni.ac.rs (S.V.); nikola.i.mitrovic@elfak.ni.ac.rs (N.M.); goran.ristic@elfak.ni.ac.rs (G.R.); danijel.dankovic@elfak.ni.ac.rs (D.D.); 3 Institute of Solid State Physics, Bulgarian Academy of Sciences, Tzarigradsko Chaussee 72, 1784 Sofia, Bulgaria; paskaleva@issp.bas.bg (A.P.);

**Keywords:** VDMOS, recovery, reliability, irradiation, NBTI stress, self-heating

## Abstract

This study investigates the effects of negative bias temperature (NBT) stress and irradiation on the threshold voltage (*V*_T_) of p-channel VDMOS transistors, focusing on degradation, recovery after each type of stress, and operational behavior under varying conditions. Shifts in *V*_T_ (Δ*V*_T_) were analyzed under different stress orders, showing distinct influence mechanisms, including defects creation and their removal and electrochemical reactions. Recovery data after each type of stress indicated ongoing electrochemical processes, influencing subsequent stress responses. Although the Δ*V*_T_ is not particularly pronounced during the recovery after irradiation, changes in subthreshold characteristics indicate the changes in defect densities that affect the behavior of the components during further application. Additionally, the findings show that the Δ*V*_T_ during the NBT stress after irradiation (up to certain doses and conditions) remains relatively stable, but this is the result of a balance of competing mechanisms. A subthreshold characteristic analysis provided a further insight into the degradation dynamics. A particular attention was paid to analyzing Δ*V*_T_ with a focus on predicting the lifetime. In practical applications, especially under pulsed operation, prior stresses altered the device’s thermal and electrical performance. It was shown that self-heating effects were more pronounced in pre-stressed components, increasing the power dissipation and thermal instability. These insights additionally highlight the importance of understanding stress-induced degradation and recovery mechanisms for optimizing VDMOS transistor reliability in advanced electronic systems.

## 1. Introduction

The serial production of electronic components and the emphasis on their development and application have led to special attention being paid to the reliability of components. Widening the range of applications of all electronic components, including metal oxide semiconductor (MOS) transistors, has resulted in these components being used under different conditions and in different environments (from extremely low to extremely high temperatures, in the presence of various types of irradiation, magnetic fields, etc.). In terms of application, there are a number of topics open for research related to the theory of component failure, critical operating conditions and lifetime estimation. Therefore, in some cases it was necessary to restructure and/or redefine the component development process, as well as the method of application, for possible changes that would give optimal results in a certain set of normal operating conditions.

It is well known that the research and development of electronic components goes through several stages. The phase that comes at the end of development is the component testing and component reliability analysis. The reliable operation of a component is defined as an operation within a set range of parameters, under certain conditions, for a certain period of time. Under the influence of some external conditions or long exposure to certain phenomena, the parameters of the component go out of the intended range. This moment is usually defined as a parametric failure of the component, and it means that the component no longer functions reliably. In many cases, components are subjected to harsh operating conditions during testing, all with the intention of accelerating the degradation process and assessing their lifetime. Several lifetime models have been proposed in the previous period, and particular lifetime models for power vertical double-diffused MOS (VDMOS) transistors have recently been the subject of intense research [1].

Due to their specific characteristics, VDMOS power transistors are widely used in both commercial and special-purpose applications. Some of the applications are in various branches of industry, such as the automotive industry or the aerospace industry, in switching power supplies, in audio amplifiers, etc. The main physical phenomena that significantly affect the change in the characteristics of VDMOS transistors and the shortening of their period of reliable operation are instabilities caused by voltage–temperature stresses with negative gate polarization (negative bias temperature instability—NBTI). In previous years, numerous research studies (Table 1) were conducted studying the essential mechanisms of NBTI effects on MOS transistors, to develop new measurement methods to estimate these effects and to decompose the individual degradation effects of components. In terms of the practical application, the work of these components in the pulse regime is much more significant; so in the last decade, these research studies have been dominant. During the pulsed mode, there are sequences throughout which gate voltage is not applied, and due to the influence of elevated temperature, the recovery phenomenon occurs.

Moreover, due to their possible application in harsh environments, there were also considerable research studies related to the influence of irradiation on VDMOS power transistors. It was observed that these components are very sensitive to ionizing irradiation, which severely degrades the threshold voltage. Finally, in real operating conditions, the component is exposed to the simultaneous or successive action of NBT stress and irradiation, so these research studies are becoming more and more important. The results shown in Table 1 clearly confirm this.

**Figure 1:**
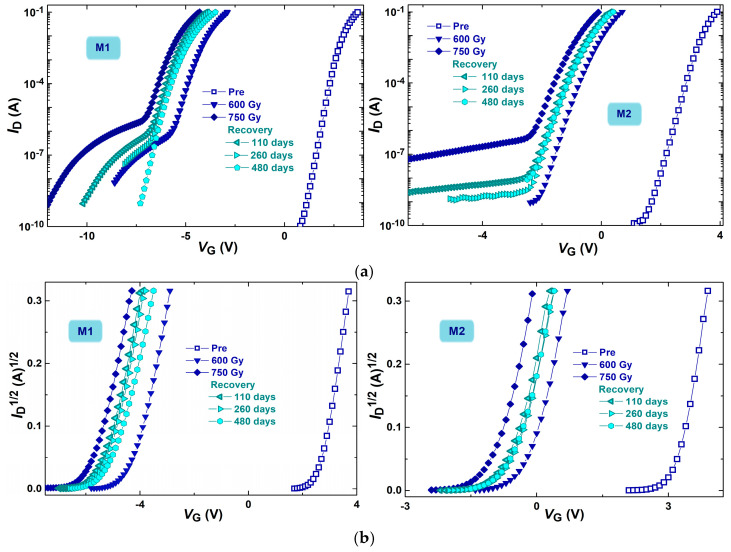
Subthreshold (**a**) and above-threshold (**b**) transfer characteristics of n-channel power VDMOSFETs (of two different manufacturers—M1 and M2) during long-term recovery after applied irradiation.

It should be noted that during our previous investigations, it was observed that during the long-term recovery of irradiated devices, subthreshold characteristics that were significantly degraded can be considerably recovered. For example, Figure 1 presents the subthreshold (a) and above-threshold (b) transfer characteristics (*I*_D_-*V*_G_) of n-channel power VDMOS transistors (of two different manufacturers—M1 and M2) during long-term recovery after applied irradiation. This indicates that processes that obviously occur even during spontaneous recovery may affect the behavior of a component during following operation under different conditions.

It is apparent that after a long spontaneous recovery, the features move back approximately half the distance between the features obtained after 600 Gy and 750 Gy. Also, it can be observed that the degradation of subthreshold characteristics practically disappeared after one year. Furthermore, it can be observed that there is a certain threshold voltage increase in both types (of different manufacturers) of devices during long-term recovery. Although this increase is not significant (approximately 6.5% of the threshold voltage value after irradiation), the changes in subthreshold characteristics clearly indicate that electrochemical reactions are taking place during the spontaneous recovery at room temperature. These reactions cause changes in the oxide trapped charge and interface traps [2,3,4], what can lead to threshold voltage changes. These changes may affect the operation of the device during further application and thus its reliability. This is the reason for the additional investigation of the threshold voltage behavior during the recovery after different stress conditions that is performed in this paper.

**Table 1:** The papers in which different aspects of MOS transistor research were performed.

	Different Aspects of Research				
Papers (First Author and Year)	Irradiation	NBTI Stress	Recovery	Activation Energy	Lifetime
Sun et al. in 2011 [5]	X		X		
Liu et al. in 2023 [6]	X				X
Li et al. in 2023 [7]	X				X
Liu et al. in 2024 [8]		X		X	
Wang et al. in 2023 [9]		X			X
Irrera et al. in 2024 [10]		X	X		
Zhao et al. in 2024 [11]		X		X	
Rinaudo et al. in 2024 [12]				X	
Biswas et al. in 2024 [13]		X			
Steinmann et al. in 2024 [14]		X			
Thakor et al. in 2024 [15]			X		X
Bonaldo et al. in 2024 [16]	X		X		X
Wang et al. in 2024 [17]		X			X
Contamin et al. in 2024 [18]		X	X		
Ghosh et al. in 2024 [19]			X		
Singh et al. in 2023 [20]			X		X
Li et al. in 2024 [21]		X			X
Liu et al. in 2024 [22]		X			X

In particular, back-up devices in an irradiation environment are exposed to the irradiation, and later under normal operating conditions, they are practically exposed to the NBT stress. This implies the importance of examining irradiation effects on the following bias temperature stress of the same devices. Due to technical limitations, it was not possible to apply both effects at the same time, so successive stress was applied to p-channel power VDMOS transistors, as presented in previous research [3,23].

Even though NBTI and irradiation effects in power MOS devices have been widely studied, they have usually been investigated separately from each other. It can be found that some studies have explored the impact of elevated temperatures on irradiation responses to better understand MOS device behavior in real irradiation environments [24,25], but those studies primarily focused on p-channel devices irradiated and/or annealed under positive gate bias. Also, investigations of successive stress were performed on p-channel power VDMOS transistors irradiated, up to only one dose, under positive, negative, or without gate bias [3,26,27]. Also, irradiation and annealing effects in NBT-stressed devices were investigated [28], as well as static and pulsed NBT effects in irradiated devices [23].

The approach of successively NBT-stressed and irradiated components allows the investigation of how one type of stress affects devices that have already been subjected to another type. Considering that devices in real applications operate under a negative gate voltage (*V*_G_), this paper presents the results of the performed experiment, in which p-channel power VDMOS transistors were irradiated under the negative gate bias or without the gate bias, up to four different dose levels.

An additional investigation of the spontaneous recovery and analysis of the underlying mechanisms was also performed, in order to elucidate their effects to further operation. On the basis of obtained results during the experiment, an analysis of reliable operation time prediction was accomplished. In addition, an analysis of self-heating effects in the investigated devices was conducted, considering that these effects may have an effect on device function [29,30].

## 2. Experimental Process

In order to conduct a complete examination of the irradiation and bias temperature stressing effects in p-channel transistors, the examined power VDMOS transistors were exposed to several stress and recovery conditions. It is important to highlight that the experiment was structured with alternating phases of stressing and transistor characteristic measurements at predefined time intervals. Moreover, a special care was taken throughout the experiment to handle the components properly, preventing electrostatic discharge and ensuring that the components remained functional until the experiment concluded.

In the conducted experiment, p-channel power VDMOS transistors, commercially available under the code IRF9520, were used as the test components [31]. These transistors are manufactured using the standard poly-Si technology and have a gate thickness of nearly 100 nm. The components are encapsulated in TO-220 plastic casings, contain 1650 cells, and feature a hexagonal configuration. The maximum current they can handle is 6.8 A, while the threshold voltage measured before the experiment was about *V*_T_ = −3.6 V. It is important to note that the initial threshold voltage may be different, depending on the manufacturer. Also, we realized some experiments with different components, with a different initial threshold voltage, in order to confirm the reproducibility of the results. In our previous studies, the results of various experiments in which components with a different initial threshold voltage were investigated can be found.

To perform this experiment, a power supply is required to deliver the appropriate static gate voltage of −45 V. Given that the thickness of the gate oxide of the investigated devices is 100 nm, the resultant electric field is 4.5 MV/cm, which corresponds to values of electric fields that cause negative bias temperature instabilities in conjunction with elevated temperatures. Another part of the setup consists of the heating chamber, which allows the temperature to be set at *T* = 175 °C. This temperature was chosen given that it corresponds to the temperatures at which NBT effects typically occur in p-channel MOSFETs operating under negative gate oxide fields. Namely, NBTI typically occur when negative gate oxide fields in the range of 2–6 MV/cm, at elevated temperatures in the range of 100–250 °C, are applied. The third part of the experimental setup includes the source measurement unit (SMU) Keysight B2901A [32]. The control of the SMU was efficiently managed via a computer interface.

In addition to the NBT stress, the irradiation process was conducted. During irradiation, a *V*_G_ of −10 V was applied to the transistors in previously selected groups, while zero *V*_G_ was applied to the remaining groups. All samples were exposed to Co-60 gamma rays at a dose rate of 8.3 mGy(SiO_2_)/s. The entire irradiation procedure took place at the Metrological Laboratory of the Institute for Nuclear Sciences in Vinča, Serbia. The selected total doses were based on data regarding the typical dose that MOS transistors in communication satellites operating in lower orbits can absorb under normal conditions [33].

**Figure 2:**
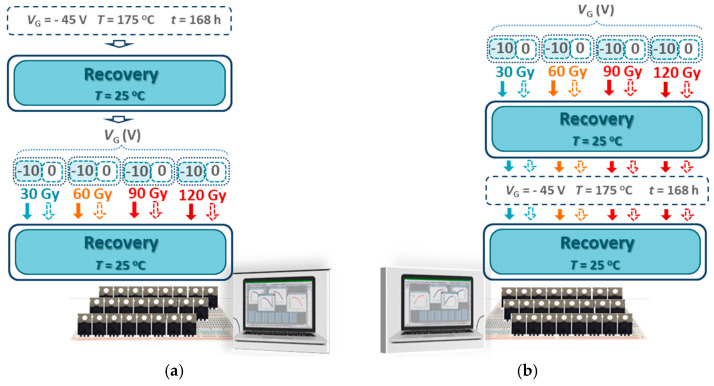
Schematic representation of the steps involved in both parts of the experiment, during which the components were subjected to (**a**) NBT stress followed by irradiation and (**b**) irradiation followed by NBT stress.

The experiment, conducted using adequate equipment, was divided into two main parts. Figure 2 shows a schematic representation of all the steps involved in both parts of the experiment. Figure 2a shows the first part of the experiment, during which the components were subjected to NBT stress followed by irradiation. During this NBT stress, the temperature in the heating chamber was held at 175 °C, with the gate biased at −45 V and the source and drain grounded for all components. The components were exposed to NBT stress for 168 h, because this duration corresponds to the conclusion of the second phases of threshold voltage shifts, which follow the known power law *t*^n^ [3,28]. After allowing a spontaneous recovery at room temperature, the components were divided into four groups, which were irradiated up to total doses of 30 Gy, 60 Gy, 90 Gy and 120 Gy, respectively. The irradiation of components from the three groups irradiated to higher doses was stopped after every absorbed 30 Gy. Before irradiation, each group was split into two subgroups. The first subgroup from each group was irradiated with a gate voltage of −10 V, while the second subgroup was irradiated without any gate voltage, with all terminals grounded.

Figure 2b shows the second part of the experiment, during which the components were subjected to irradiation followed by NBT stress. Similarly to the first part of the experiment, before the irradiation the components were divided into four groups, which were irradiated up to the full doses of 30 Gy, 60 Gy, 90 Gy and 120 Gy, respectively. Also, the irradiation of components from the three groups irradiated to higher doses was stopped after every absorbed 30 Gy. Each of these groups was split into two subgroups, where the first subgroup was irradiated with a gate voltage of −10 V, while the second subgroup was irradiated without any gate voltage, with all terminals grounded. After spontaneous recovery at room temperature, the components of all eight subgroups were exposed to NBT stress for 168 h. The same as in the first part of the experiment, the temperature in the heating chamber was held at 175 °C, with the gate biased at −45 V and the source and drain grounded for all components.

During both parts of the experiment, the experimental process was interrupted, at certain (previously defined) intervals, in order to perform the electrical characterization. This characterization was carried out by measuring the transfer characteristics *I*_D_-*V*_G_.

## 3. Transfer Characteristics

To detect the transfer characteristics, the source measurement unit Keysight B2901A system, was employed. It should be noted, it is essential to remove the applied stress voltage or irradiation from the device undergoing testing before conducting *I-V* measurements. Subsequently, the threshold voltage is determined from the measured transfer characteristics (the above part).

**Figure 3:**
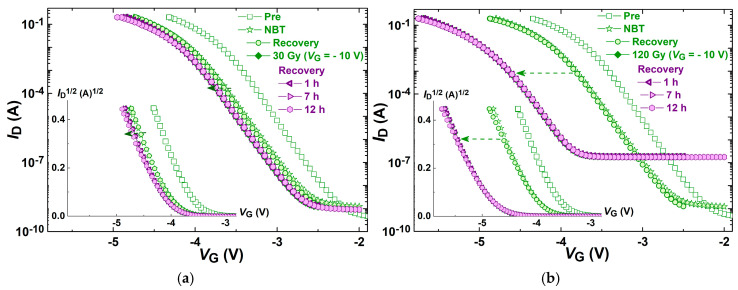
Components’ transfer characteristic shifts induced by NBT stress followed by irradiation with a gate voltage of −10 V (**a**) up to 30 Gy and (**b**) up to 120 Gy, and transfer characteristics after the spontaneous recovery that tailed both stresses.

**Figure 4:**
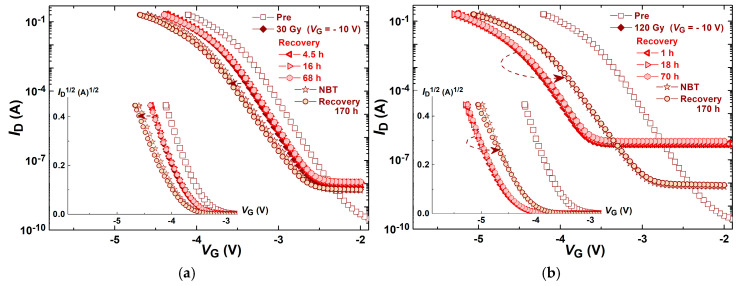
Components’ transfer characteristic shifts induced by irradiation with a gate voltage of −10 V (**a**) up to 30 Gy and (**b**) up to 120 Gy, followed by NBT stress, and transfer characteristics after the spontaneous recovery that tailed both stresses.

Figure 3 represents the transfer characteristic shifts of components induced by negative bias temperature stress followed by irradiation, while Figure 4 represents components transfer characteristic shifts induced by irradiation followed by negative bias temperature stress. Subthreshold transfer characteristics are presented in these figures, while the above-threshold parts of the transfer characteristics are presented in inserted figures.

Shifts in the component transfer characteristics induced by the NBT voltage followed by irradiation with a gate voltage of −10 V up to 30 Gy are shown in Figure 3a and up to 120 Gy in Figure 3b. Also, transfer characteristics after the spontaneous recovery that tailed both stresses are presented in these figures. It can be seen that NBT caused a significant shift along the *V*_G_ axis toward more negative values and somewhat of a decrease in the slope, and that spontaneous recovery after NBT led to a slight decrease in the slope of the transfer characteristics. The irradiation up to 120 Gy caused a significant shift along the *V*_G_ axis, as expected. The spontaneous recovery after the irradiation caused a decrease in the transfer characteristics slope, which can be observed in both parts of the characteristics (subthreshold and above-threshold), in both subgroups of components—irradiated up to 30 Gy, as well as up to 120 Gy.

The shift in transfer characteristics for components irradiated up to 30 Gy would be more visible with a smaller range of the gate voltage value, but the same range was used for all examples shown in order to compare the shifts in characteristics.

In Figure 4a,b, transfer characteristic shifts of NBT-stressed components that were previously irradiated, with a gate voltage of −10 V, up to 30 Gy and up to 120 Gy are presented, respectively. Also, these figures present the transfer characteristic shifts after the spontaneous recovery that tailed both stresses. As usual, irradiation up to 120 Gy caused a more significant shift along the *V*_G_ axis, toward more negative values. Similarly to the previous experimental procedure, the spontaneous recovery after the irradiation caused a decrease in the transfer characteristics slope, which can be observed in both parts of the characteristics (subthreshold and above-threshold), in both subgroups of components—stressed up to 30 Gy, as well as up to 120 Gy. However, the shifts in transfer characteristics during the following NBT stress are completely different in these subgroups. In devices previously irradiated up to 30 Gy, the NBT stress induced a further shift of characteristics along the *V*_G_ axis, toward more negative values, as is shown in Figure 4a. Conversely, in devices previously irradiated up to 120 Gy, the NBT stress induced an opposite shift of characteristics along the *V*_G_ axis, a shift toward less negative values, as is shown in Figure 4b. Nevertheless, the subsequent spontaneous recovery after the irradiation caused a decrease in the transfer characteristics slope, which can be observed in both parts of the characteristics (subthreshold and above-threshold), in both subgroups of components—stressed up to 30 Gy, as well as up to 120 Gy.

The degradation in transfer characteristics is significantly higher in devices irradiated up to 120 Gy, and leakage current is somewhat more pronounced in devices which were not previously NBT stressed. Also, NBT after irradiation up to 30 Gy and 120 Gy causes a lowering of leakage current in both cases, at the approximately the same value. The observed transfer characteristic variations in the tested components are caused by electrochemical processes, during which the oxide trapped charge (*N*_ot_) and interface traps (*N*_it_) are created. The creation of these defects causes the changes in the parameters of the transistors, of which the threshold voltage shift is the most significant and could be a limiting factor for normal operating conditions. The contributions of gate oxide charge (Δ*V*_ot_) and interface traps (Δ*V*_it_) to the threshold voltage shifts can be presented as

Δ*V*_T_ = Δ*V*_ot_ + Δ*V*_it_. (1)

This represents a model used to describe changes in the threshold voltage due to various mechanisms affecting semiconductor devices, particularly field-effect transistors. Recent scientific papers describe this equation when analyzing device degradation under stress, such as the impact of interface and hole traps on the threshold voltage shift in FETs [3,8,26,30,34,35,36]. These components are often uncorrelated and arise from different physical processes, such as the carrier trapping at the oxide interface or defects in the gate dielectric. Also, this modeling framework has been applied to advanced devices like FinFETs and nanosheet transistors [21,37].

The contributions to the threshold voltage shift can be estimated by the widely used subthreshold midgap technique [38], which can further shed light on the processes occurring in the component that affect its behavior during operation in a specific environment and cause changes in the threshold voltage.

## 4. Threshold Voltage Shift and Lifetime Prediction

The threshold voltage was determined from the transfer characteristics of the investigated components, by extrapolating the linear region of the (√*I*_D_)-*V*_G_ curves and identifying the point where the line intersects the *V*_G_ axis [39]. This method is often used in advanced semiconductor and nanoelectronics research, where precision in *V*_T_ extraction is crucial for performance evaluation and optimization. Before starting the experiment, the transfer characteristics of the fresh components were measured, and the threshold voltage of each of them was determined. In order to obtain reliable data, we carefully selected components whose threshold voltages are in a narrow range (closest transfer characteristics and threshold voltage values). Although during the irradiation and NBT stress, no large spreading of the results of our measurements was observed, multiple measurements are provided, and three components (in each subgroup) were subjected to absolutely the same conditions of stress and irradiation. In order to ensure the reliability and establish the reproducibility of the observed results, 24 components were needed (irradiation up to four doses, with and without gate voltage) for each of the two parts of the experiment (NBT stress followed with irradiation and irradiation followed with NBT stress), which is a total of 48 tested components.

**Figure 5:**
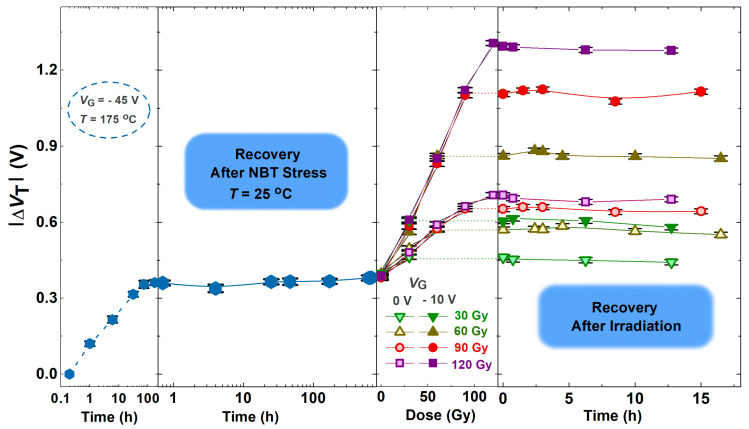
Threshold voltage shift through NBT stress followed by irradiation and during the spontaneous recovery that tailed both phases.

**Figure 6:**
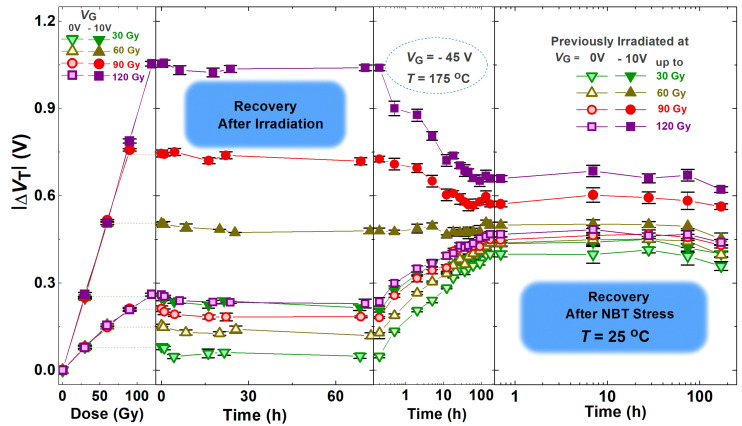
Threshold voltage shift through irradiation followed by NBT stress and during the spontaneous recovery that tailed both phases.

For better tracking of the experimental data and clarity, the shown values of Δ*V*_T_ represent the mean values of the threshold voltage shift of the components from each subgroup. In Figure 5 and Figure 6, which represent the whole first and second part of the experiment, the threshold voltage mean values of the components of all subgroups are present, as well as standard deviations.

Figure 5 shows the threshold voltage shift through NBT stress followed by irradiation, while Figure 6 shows the threshold voltage shift through the opposite order of stresses—NBT stress following irradiation. Also, in these figures are presented the threshold voltage shift during the spontaneous recovery that tailed both phases, in both order of stresses. The threshold voltage shift is presented in an absolute value because its value in p-channel VDMOS transistors is negative, and it increases in absolute value (during the irradiation or NBT stress); so, the corresponding shifts are shown as positive.

As can be seen (Figure 5), all fresh components exposed to the NBT stress practically show almost the same degradation curve of Δ*V*_T_. In numerous studies of NBT instability, there were statements that the creation of an interface trap could be a reaction-controlled mechanism. While there has been disagreement regarding the role of trapped charges in NBT instability [40,41], many studies have recommended that hole trapping mostly contributes to degradation [42,43]. This has led to the suggestion of a new charge trapping model, linking NBTI degradation to the creation of switching oxide traps. The model fits with the recovery data, which shows dispersion above a wide time range.

In Figure 6, it can be noticed that irradiating fresh devices caused significant negative shifts in the threshold voltage. These shifts increased with the total dose received and varied depending on the applied gate bias. At zero bias, the threshold voltage shift was small, but when the negative gate voltage was applied during the irradiation, the threshold voltage shift was nearly four times greater. This variation is attributed to the influence of the electric field on irradiation effects [3,26,27].

Many models for the elucidation of the mechanisms responsible for the changes in *N*_ot_ and *N*_it_ during gamma radiation and NBT stress can be found in the literature [44,45,46,47,48]. The suggested electro-chemical reactions in these models are founded on the presence of charge trap precursors in the gate oxide and at the SiO_2_-Si interface, as well as on the movement of hydrogen particles.

During irradiation, the high energy of the photons breaks the weak Si-H and Si-OH bonds in the oxide, as well as the regular bonds, and electron–hole pairs can be created. In the case of applied negative gate voltage, the electrons will be almost immediately removed through the semiconductor. The created holes can be trapped in the oxide, contributing to increase in *N*_ot_, which increases the local electric field. The increased amount of holes can participate in a chain of mechanisms that lead to the growth in *N*_ot_ and *N*_it_ [26,44]. As a consequence of these increases, there is a more pronounced increase in *V*_T_ in the case of the applied negative gate voltage.

In the case of NBT stress, the applied electric field can dissociate interfacial Si-H bonds, leading to the release of a hydrogen atom (H) and creation of *N*it [45,46,47]. The released H is very reactive and additionally can dissociate the interfacial Si-H bonds, which leads to further creation of *N*it [45,46,48]. The released H, in reaction with a hole, creates a hydrogen ion, which drifts from the interface (due to negative gate polarization) and can dissociate the Si-H bonds in the gate oxide, which contributes to an increase in *N*_ot_. All the mentioned reactions can also occur in the reverse direction, which is especially important during the recovery after the applied stress. The additional creation of *N*_ot_ can be ascribed to hole trapping at oxygen vacancy defects and at dangling Si-H bonds [48].The trapped *N*_ot_ progressively decreases the local electric field near the interface, causing the slowing down of its creation during further stress. Moreover, *N*_ot_ may be transformed into *N*_it_, especially in the later stage of the stress.

Most of the reactions that occur during NBT stress can follow either a forward or reverse direction. In the case of fresh devices NBT, stress reactions dominantly a follow forward direction, leading to defect creation. However, in the case of previously applied irradiation, the direction of each reaction can change with the different NBT stress pretreatment leading to defect creation (a forward direction of reactions) or to its passivation (a reversed direction of reactions). In the case of recovery, the reverse direction of reactions is much more likely.

**Figure 7:**
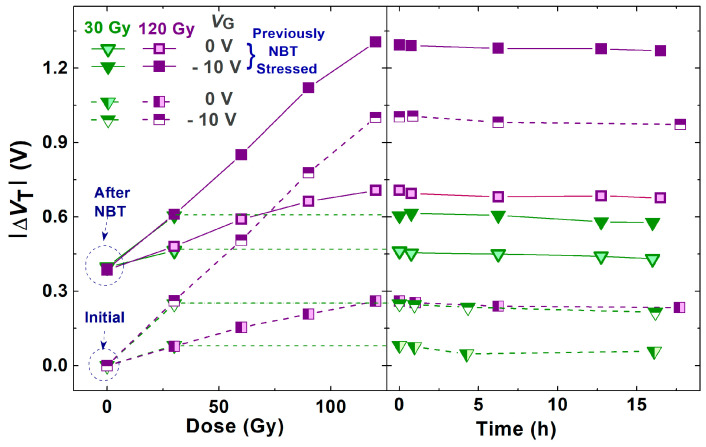
Threshold voltage shift through irradiation and the spontaneous recovery of fresh and previously NBT-stressed components.

**Figure 8:**
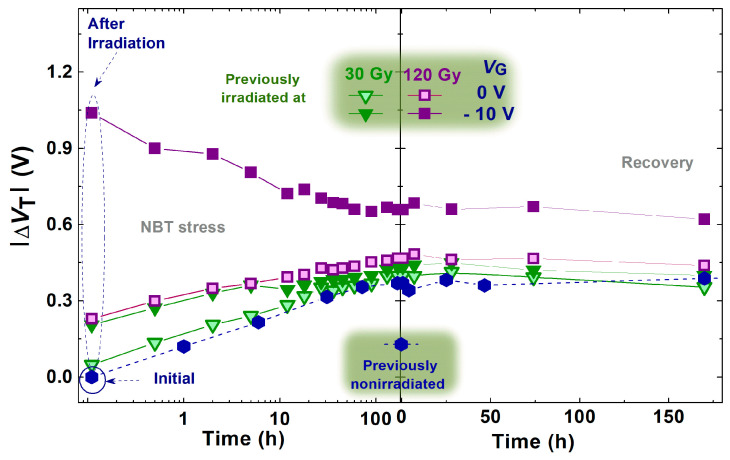
Threshold voltage shift through NBT stress and the spontaneous recovery of fresh and previously irradiated components.

Although the changes in Δ*V*_T_ during spontaneous recovery after the negative bias temperature stress, as well as after irradiation, do not seem especially notable (Figure 7 and Figure 8), the slight shift and changes in the slope of transfer characteristics (Figure 3 and Figure 4) indicate that even during spontaneous recovery, electrochemical processes are still taking place. This cannot be neglected, because the electrochemical processes that occurred may affect Δ*V*_T_ during the following stress. Namely, during the next irradiation, the changes in Δ*V*_T_ (for all doses in both cases—irradiated with or without polarization) are somewhat smaller than the corresponding changes in devices which were not stressed before the irradiation, as can be seen in Figure 7. Especially, the NBT stress after irradiation is significantly affected by the previous irradiation (Figure 8), which requires additional analysis.

Namely, it can be noticed in Figure 6 that the irradiation significantly affected the threshold shift during the later NBT stress. In all components that were irradiated with no gate voltage applied and in those that were irradiated with a negative gate voltage applied to the components irradiated to the lowest dose, the NBT stress caused a rise in the threshold voltage shift. Quite the opposite, in components irradiated with a negative gate voltage applied to the components irradiated to the two highest doses, the NBT stress caused a decrease in the threshold voltage shift. This decrease was more pronounced for the highest dose—up to 120 Gy. However, in the components irradiated up to 60 Gy, the NBT stress did not cause significant changes of the threshold voltage shift.

**Figure 9:**
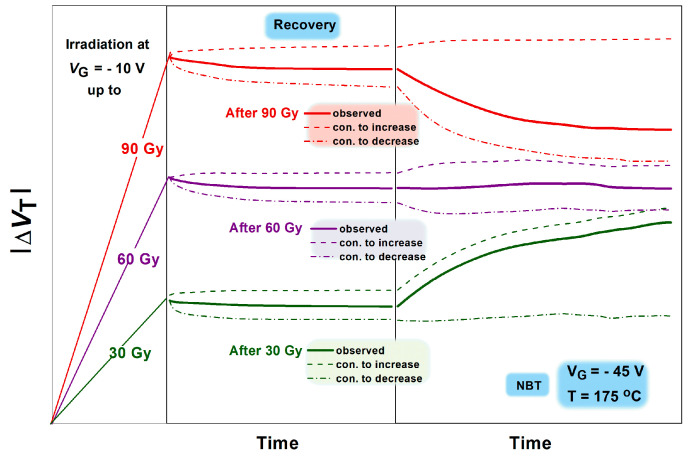
Schematic illustration of two parts—contributions to the increase and decrease in threshold voltage shift, which are caused by the mechanisms of activation of electrochemical reactions and annealing.

These behaviors of the threshold voltage may be explained by two mechanisms: the activation of electrochemical reactions contributing to NBTI, leading to additional oxide trapped charge and interface trap creation, and the annealing of the irradiation-induced trapped charge due to the negative voltage applied and an elevated temperature of 175 °C used during the NBT stress. These two mechanisms contribute to an increase and decrease in the threshold voltage shift, which is schematically presented in Figure 9. For devices irradiated to all doses with no gate voltage and irradiated to the lowest dose with gate voltage, the irradiation-induced defects were relatively few, while a significant number of defect precursors remained. Consequently, the subsequent NBT stress primarily resulted in additional defect creation, leading to a further increase in the threshold voltage shift. In contrast, for devices irradiated with gate voltage up to the highest doses, the number of irradiation-induced defects was much higher, and during the NBT stress, the annealing of these defects dominated over new defect creation, resulting in a decrease in the threshold voltage shift. In this case, the effects induced by the elevated temperature have prevailed. It should be mentioned that in Figure 9 is presented a schematic illustration of the observed values (solid line), as well as two contributions to the increase and decrease in threshold voltage shift, which are caused by the mechanisms of activation of electrochemical reactions. It is obvious that during the NBT stress, where subsequently the irradiation with gate voltage has been applied, in those devices irradiated up to 90 Gy the contribution to the decrease is dominant, while in the devices irradiated up to 30 Gy the contribution to the increase is dominant. Simultaneously, in the devices irradiated up to 60 Gy, the contributions to the decrease and increase are in balance

Similarly, although the changes in Δ*V*_T_ during the spontaneous recovery after NBT stress and irradiation appear relatively minor (Figure 7 and Figure 8), the slight shifts along the *V*_G_ axis caused by changes in oxide trapped charge, and variations in the slope of the transfer characteristics caused by interface traps (Figure 3 and Figure 4), suggest that electrochemical processes may still be occurring during the recovery, and that these processes can be in balance. However, the created and annealed oxide trapped charge and interface traps cannot be overlooked, as they may have an influence during the subsequent stresses.

Obviously, the electrochemical processes that occurred during the spontaneous recovery can influence the behavior of the components during the following operating conditions. Regarding this, an investigation of component behavior during all parts of the experiment, even through stabilization–recovery, may be of high importance. This statement can be additionally supported by analyzing changes in subthreshold characteristics, which are caused by changes in the oxide trapped charge and interface traps.

In Figure 3, it can be observed that the NBT voltage caused a slight increase in the leakage current, but the subsequent irradiation caused a significant increase in the leakage current, more than two orders of magnitude (in devices irradiated up to 120 Gy). On the other hand, the irradiation of fresh components caused an even more significant increase in the leakage current (Figure 4), more than three orders of magnitude (in devices irradiated up to 120 Gy). However, the subsequent NBT step led to a two orders of magnitude decrease in the leakage current. So in this case, after both stresses, the leakage current is more than one order of magnitude lower than after the opposite sequence of stresses.

As has been mentioned, in Figure 3 and Figure 4 it can be seen that the irradiation has created the degradation of subthreshold characteristics with a high negative shift at low current (in the midgap current region) which can be attributed to a large positive oxide charge. At the same time, degraded subthreshold characteristics can manifest different slopes at different voltage positions (like in Figure 1a), which can be attributed to different interface trap densisties at different energies within the silicon bandgap. Namely, the exponential dependence of the drain current *I_D_* on the surface potential ϕ_s_, and the capacitive voltage divider in the gate–oxide–silicon region, give us the expression of the subthreshold slope:

(2) $$\frac{d({logI}_{D})}{dV_{GS}}=\frac{d({logI}_{D})}{d\varphi_{S}}\cdot \frac{d\varphi_{S}}{dV_{GS}}=\frac{1}{\frac{kT}{q}ln10}\cdot \frac{1}{1+\frac{{qD}_{it}}{C_{ox}}}$$

where *V_GS_* is the voltage applied to the gate, *ϕ*_s_ is the surface potencial, k is the Boltzmann constant (1.380649·10^−23^ m^2^ kg s^−2^ K^−1^), *T* is the absolute temperature (in K) and *C*ox is the capacitance of oxide (F/cm^2^), while *D_it_* is the value of the interface trap density per unit of surface area and unit of surface potential (cm^−2^ V^−2^), *q* is the leementary charge, *I*_D_ is the drain current and *V*_GS_ is the gate source voltage.

Going upward from the midgap voltage point to the threshold voltage, the subthreshold slope decreases, which can be explained as a localization of energy trap levels close to the middle of the silicon bandgap. During the recovery phase, the oxide charge decreases, and at the same time, the localized density of interface traps also decreases, i.e., traps are redistributed or energy relaxed.

The behavior of the subthreshold characteristics during the irradiation of the devices presented in Figure 3 and Figure 4 expresses high linearity, which can be mathematically modeled. Namely, the same total dose produces a lower negative threshold voltage shift, whereas in the midgap region the negative shift is very expressed.

These behaviors of subthreshold characteristics, and especially the leakage current, require additional analyses, given that structure of VDMOS transistor is very complex, and in that case thermally activated electrochemical mechanisms could play a significant role. Changes in charge trapped in the VDMOS transistor during applied stresses can notably affect the path of the leakage current, what requires futher analysis.

**Figure 10:**
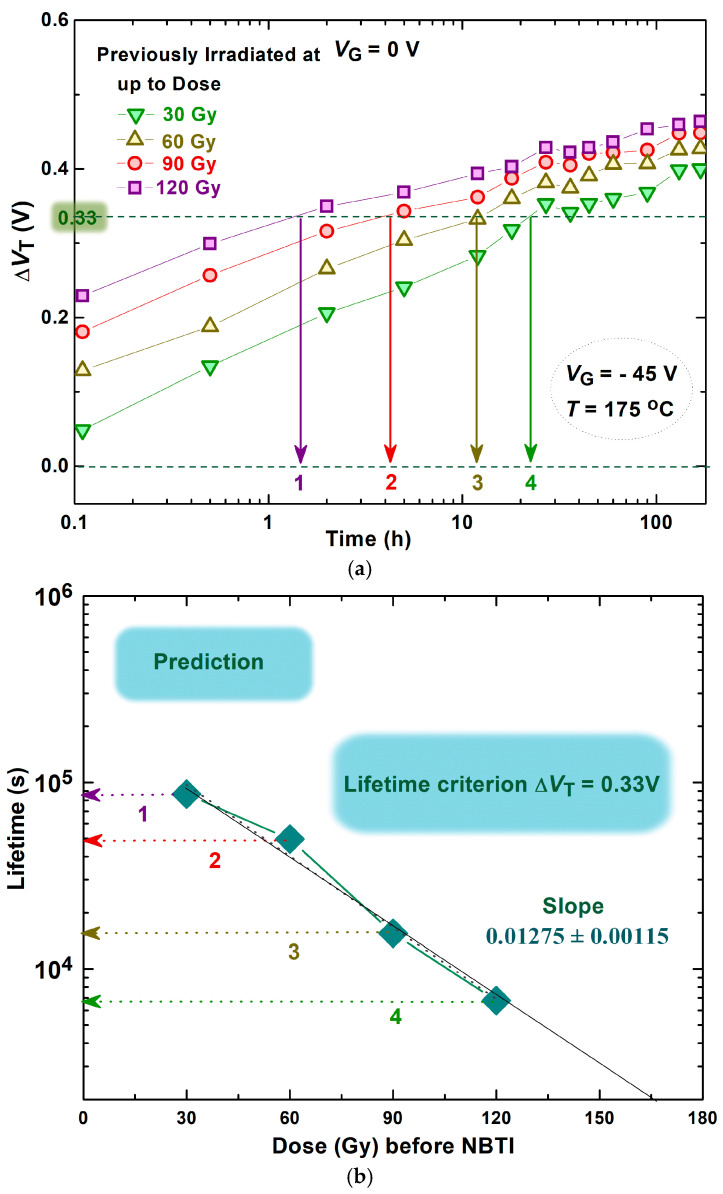
Changes in threshold voltage shift through NBT stress, after irradiation with no gate voltage applied up to four achieved doses (**a**) and lifetime prediction (**b**).

Also, an additional analysis was aimed at predicting the lifetime of VDMOS transistors. As has been mentioned, when a component’s parameter deviates from its intended range due to external conditions or prolonged exposure to certain phenomena, it is considered a parametric failure. At this point, the component is deemed no longer reliable in its operation. For the tested components, it was adopted that the changes in the threshold voltage shift of 0.33 V are close to the critical value, as shown in our previous investigation [49]. This value can be noticed in Figure 10a, which presents the changes in threshold voltage shift through the NBT stress, subsequent to irradiation with no gate voltage applied, which previously were irradiated up to four total doses. Based on these behaviors of the threshold voltage and their intersection with the value of 0.33 V, the times when changes in the threshold voltage achieve this value were determined. These times, so-called experimental values of lifetimes, versus the achieved irradiation dose are presented in Figure 10b. Using these results, the lifetime could be predicted for any value found in real operating conditions.

Although the results obtained during the static NBT stress can be used to predict a reliable operation time, it should be noted that from the point of view of a practical application, the operation of these components in the impulse mode is of a greater importance.

The switching power supply, automotive and space sectors are recognized to be the main users of VDMOS devices [49,50,51]. In order for VDMOS devices to operate as effective switches, most of these applications depend on their exceptional switching capabilities. For that reason, the controlling signal usually has a pulsed waveform. The devices are suitable for various circuit applications operating at standard frequencies on the order of MHz. The transistor’s on*-* and off-time are determined by the properties of the controlling signal, including the duty cycle, time of rising and falling edge. The VDMOS transistor operates as a closed switch when the voltage of the controlling signal exceeds *V*_T_; otherwise, it operates as an open switch. During the operation, it is observed that the threshold voltage of the VDMOS transistor varies as a result of self-heating [52]. Taking into account all the factors, the aim of this experimental segment was to assess how prior stresses influence actual operating conditions.

**Figure 11:**
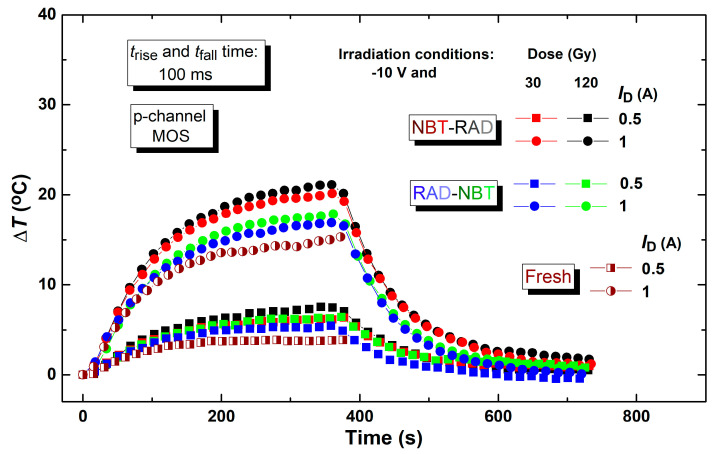
The temperature change of two stressed groups of components, NBT-RAD and RAD-NBT, with four different drain currents.

Figure 11 shows how the temperature changes over time but in specific conditions. One of the goals was to simulate operating settings that are as close as possible to those existing in some applications. So, the threshold voltage shifts for the components used in this part of the experiment were analyzed in the previous section, where the results of the components that were exposed to two combinations of stress, negative bias temperature stress and irradiation, are presented. Namely, the results for p-channel VDMOS transistors, each exposed to different pre-stress histories, were monitored. It should be mentioned that the components were exposed to a long spontaneous recovery at room temperature, for several years. The initial set consisted of components that had not been subjected to any earlier stress. For all those sets of components, the self-heating effects were monitored.

While heating, all the component groups had been exposed to pulsed signals characterized by parameters typical of switching power supplies: a frequency of 1 Hz, time of rising and falling edge of 100 ms and duty cycle of 50%. Additionally, currents of 0.5 A and 1 A, denoting the active load, were incorporated into the drain circuit. The subsequent cooling under all conditions was measured, alongside a more detailed analysis of the heating.

The curves in Figure 11 exhibit a temperature dependence that remains varying throughout time. The rise in the chip’s temperature is primarily attributed to the consequences of power dissipation. Each pulse transition induces further stress on the tested components. Temperature increases over the period of each edge and drops towards thermal equilibrium during the time of pulse absence. The temperature rises consistently with a greater number of pulses [53]. Furthermore, previously stressed samples exhibit alterations in threshold voltage, characterized by an elevation in the absolute threshold voltage value. This prolongs the duration of channel opening at a constant gate voltage, resulting in the current passing through increased resistance in stressed devices for extended intervals, hence causing the power dissipation.

In Figure 11 which illustrates the variations in temperature over time within actual operating conditions, it can be noted that fresh components exhibit a lower temperature increase than previously stressed components. As for the stressed components, it is clearly observed that the increase in temperature is greater for those for which the irradiation was the second stress. And among the components that are subjected to the same order of stress, it is clear that the increase in temperature is in those that were irradiated up to higher total doses. This is more noticeable as the applied current is higher.

## 5. Conclusions

This study presents a comprehensive analysis of the threshold voltage shifts in p-channel VDMOS transistors under combined irradiation and negative bias temperature stress. The experimental results highlight the intricate interplay between oxide charge creation, interface trap formation and annealing mechanisms, which jointly influence the degradation and recovery of these devices. Spontaneous recovery, though showing relatively minor shifts in the threshold voltage, indicates ongoing electrochemical reactions and charge redistribution, which may influence subsequent stress responses. Additionally, subthreshold characteristics reveal degradation effects associated with oxide charge and interface traps, providing further evidence of the complex physical mechanisms at play. The results demonstrate that the sequence of stress application critically influences the threshold voltage behavior. Components irradiated prior to NBT stress show varying threshold voltage shifts depending on the irradiation dose and gate voltage applied. At lower doses, the NBT stress leads to an increase in threshold voltage due to additional defect creation, while at higher doses, defect annealing dominates, resulting in a reduction in threshold voltage shift. These phenomena highlight the interplay between charge trapping, interface trap formation and electrochemical processes, which can shift in dominance depending on the stress conditions. Dynamic stress tests under pulsed operation conditions emphasize the practical implications of the observed degradation mechanisms. Devices with prior stress histories exhibit more pronounced self-heating effects, leading to increased power dissipation and potential long-term reliability issues. These findings underscore the importance of understanding threshold voltage dynamics under real-world operating conditions. The study also provides a framework for predicting device lifetimes based on critical threshold voltage shifts and highlights the relevance of stress order and recovery phases in ensuring the reliable operation of VDMOS transistors in demanding applications such as switching power supplies, automotive systems and space technology. Further exploration of stress-induced degradation under pulse-mode operation could provide additional insights for optimizing device performance and reliability in these areas.

## Data Availability

The original contributions presented in the study are included in the article, further inquiries can be directed to the corresponding authors.
